# Synthesis of Novel Compounds as New Potent Tyrosinase Inhibitors

**DOI:** 10.1155/2013/207181

**Published:** 2013-10-22

**Authors:** Hooshang Hamidian

**Affiliations:** Department of Chemistry, Payame Noor University (PNU), P.O. Box 19395-3697, Tehran, Iran

## Abstract

In the present paper, we report the synthesis and pharmacological evaluation of a new series of azo compounds with different groups (1-naphthol, 2-naphthol, and *N,N*-dimethylaniline) and trifluoromethoxy and fluoro substituents in the scaffold. All synthesized compounds (**5a–5f**) showed the most potent mushroom tyrosinase inhibition (IC_50_ values in the range of 4.39 ± 0.76–1.71 ± 0.49 *µ*M), comparable to the kojic acid, as reference standard inhibitor. All the novel compounds were characterized by FT-IR, ^1^H NMR, ^13^C NMR, and elemental analysis.

## 1. Introduction

Tyrosinase inhibitors are clinically useful for the treatment of skin diseases associated with melanin hyperpigmentation and applied in cosmetics for whitening and depigmentation after sunburn. Melanin is a heteropolymer of indole compounds and is produced inside melanosomes by the action of the tyrosinase enzyme on the tyrosinase precursor material in melanocytes. It has recently been discovered that some other factors such as metal ions and the TRP-1 and TRP-2 enzymes also contribute to the production of melanin. However, tyrosinase plays a critical role in the regulation of melanin biosynthesis. Therefore, many tyrosinase inhibitors that suppress melanogenesis have been widely studied with the aim of developing preparations for the treatment of hyperpigmentation [1–5].

It is well known that azo compounds are the most widely used class of industrial synthesized organic compounds because of their versatile usage in different fields, like dyeing textile fiber, biological–pharmacological activities, and advanced usage in organic synthesis [6–13]. In recent years, the fabrication of azo dyes has been widely investigated because of their unique industrial usages in hypnotic drugs, in living cells, in detecting cancer and owning pharmacological and biological activities [14–17].

In this research, we synthesized number of new azo compounds and studied chemical structures. Also we evaluated inhibitory effect on tyrosinase, melanin production inhibition and cytotoxicity of new compounds.

## 2. Results and Discussion

**Scheme 1:**
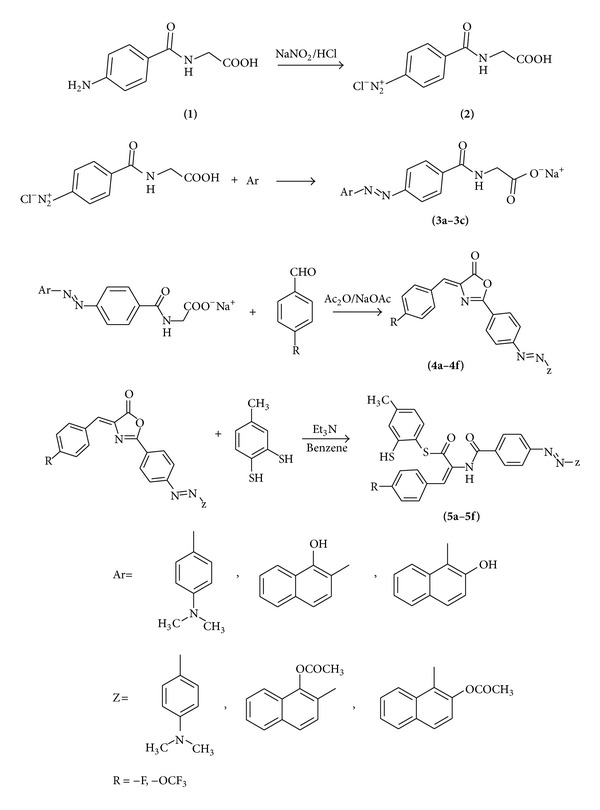
Synthesis of compounds **5a–5f**.

Diazonium salts could react readily with nucleophiles as aromatic compounds containing amino or hydroxyl group, which have been widely researched and applied for the preparation of molecules with importance for both academic and industrial applications. The solution of 4-amino hippuric acid and sodium nitrite in a 2.5% sodium carbonate was diazotized by slow addition of conc. HCl at 0°C. A yellow precipitate diazonium salt **(2)** was formed. The coupling components (*N,N*-dimethylaniline, 1-naphthol, and 2-naphthol) were added to diazonium salt of 4-aminohippuric acid. Azo compounds **3a–3c** were produced in good yields. Diazonium salt was coupled with the *para*-position of the amine group, 2-position of hydroxyl group in 1-naphthol and 1-position of hydroxyl group in 2-naphthol. Then, 4-arylidene-5(4*H*)-oxazolones **4a–4f** were synthesized by classical Erlenmeyer reaction, involving condensation of compounds **3a–3c** with 4-fluorobenzaldehyde and 4-trifluoromethoxy benzaldehyde in the presence of acetic anhydride and sodium acetate under refluxing conditions. New azo compounds **5a–5f** were prepared by the reaction of 4-arylidene-5(4*H*)-oxazolones **4a–4f** and 3,4-dithio-toluene in the presence of triethylamine at the room temperature in dry benzene (Scheme 1).

Generally, variation in color of these dyes results from the alternation in the coupling components, since the synthesized dyes which were obtained varied in color from red to brown. Compounds (**5a–5f)** were stable solids whose structures were established by FT-IR, ^1^H NMR, ^13^C NMR spectroscopy, and elemental analysis (Table 1).

**Figure 1:**
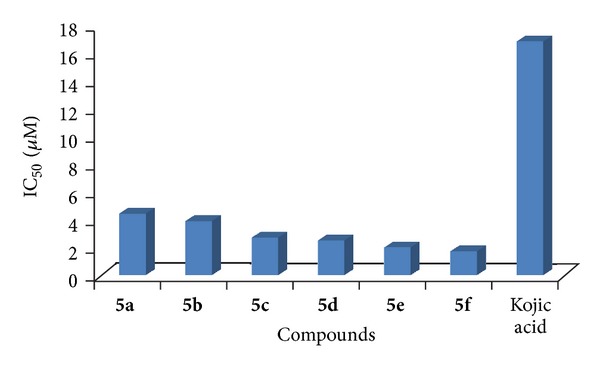
Comparative graphical presentation of the tyrosinase inhibitory potentials of the compounds **5a–5f**.

The compounds **5a–5f** demonstrated excellent in vitro tyrosinase inhibitory properties having IC_50_ values in the range of 4.39 ± 0.76–1.71 ± 0.49 *μ*M, whereas standard inhibitor, Kojic acid has IC_50_ value 16.67 ± 0.52 *μ*M (Table 2 and Figure 1).

Also inhibitions of the compounds **5a–5f** were tested on melanin production and their cytotoxicity on B16F10 mouse melanoma cells at concentrations of 20 *μ*g/mL. The results of melanin production inhibition and cytotoxicity by the compounds **5a–5f** are showed in Table 3.

Compounds **5e** and **5f** having IC_50_ values 1.98 ± 0.39 and 1.71 ± 0.49, respectively, were found to be very active members of the series, even better than standard inhibitor. 1-(2-{4-[({1-{[(4-Methyl-3-sulfanylphenyl)sulfanyl] carbonyl}-2-[4-(trifluoromethoxy)phenyl]-1-ethenyl}amino) carbonyl]phenyl}-1-diazenyl)-2-naphthyl acetate **5f** was found to be the most active one having IC_50_ = 1.71 ± 0.49 *μ*M among all tested compounds.

Comparing the activities with the structures of compounds, it turns out that the tyrosinase activity is mainly dependent on the substituent present at C-4 positions of aryl ring (-F or −CF_3_). When tyrosinase inhibitory activity of the most active compounds **5b, 5d**, and **5f** was compared with other compounds **5a, 5c,** and **5e**, it was observed that it has a 4-(trifluoromethoxy) phenyl group on the aliphatic double bond.

A decrease in the activity of compounds **5a** and **5b** as compared to compounds **5c–5f** was due to the change in the phenyl to naphthyl at azo group. This shows that extension of conjugation through an aliphatic double bond could be the prerequisite for activity rather than extension through an aromatic ring.

The least activity of compound **5a** (IC_50_ 4.39 ± 0.76 *μ*M) may be due to changing the substituent in phenyl rings present at C-4 and aromatic ring. Compound **5f** (IC_50_ 1.79 ± 0.49 *μ*M) was found to be highly active member of the present series of azo compounds. Its excellent activity may be due to the presence of naphthyl ring and the presence of a trifluoromethoxy group in phenyl ring at C-4, which meets the criteria for achieving extension of conjugation. Compound **5e** (IC_50_ 1.98 ± 0.39 *μ*M) is structurally similar to compound **5f** except where trifluoromethoxy group is replaced by fluoro. Interestingly, compounds **5a–5d** having IC_50_ values 4.39 ± 0.76, 3.86 ± 0.66, 2.68 ± 0.55, and 2.48 ± 0.88 *μ*M, respectively, showed good activity.

Compounds **5a–5f** prevented melanin production by 42.15%, 40.98%, 35.93%, 37.50%, 36.84%, and 34.74%, respectively, at concentrations of 20 *μ*g/mL. On the other hand, compounds **5a–5f** have shown moderate inhibition of melanin production. Cytotoxicity of new compounds **5a–5f** was evaluated and was defined that all compounds were less toxic (Table 3).

All synthesized azo dyes exhibited high tyrosinase inhibitory behavior. The results of mushroom tyrosinase inhibition assays indicate that the 4-trifluoromethoxy derivatives have high degrees of inhibition and 1-naphthol and 2-naphthol derivatives are better for tyrosinase inhibition than *N,N*-dimethylaniline derivatives. All synthesized azo compounds **5a–5f** showed the most potent mushroom tyrosinase inhibition, comparable to that of Kojic acid as reference standard inhibitors.

## 3. Experimental

### 3.1. General Information

All the chemicals were obtained from Merck, Fluka, and Sigma-Aldrich and were used without further purification. Melting points were measured using Thermo Fisher Scientific. IR spectra were recorded by Bruker tensor 27, FT- IR spectrophotometer. All ^1^H NMR and ^13^C NMR spectra were recorded by a Bruker 400 MHz spectrophotometer. Chemical shifts are reported in parts per million (ppm) using tetramethylsilane (TMS) as an internal standard. The mass spectra were run on a Shimadzu Qp 5050 Ex spectrometer. The microanalyses for C, H, and N were performed on Perkin-Elmer elemental analyzer. Ultraviolet-visible (UV-vis) absorption spectra were recorded on a Perkin Elmer spectrophotometer at the wavelength of maximum absorption (*λ*_max⁡_) in dimethylsulphoxide (DMSO) at the same level of concentration (1 × 10^−5 ^M).

### 3.2. Preparation of Diazonium Salt of 4-Aminohippuric Acid **(2)**

In a 125-mL erlenmeyer flask, 4-aminohippuric acid (0.01 mol) was added to 2% sodium carbonate solution (30 mL) until it was dissolved by boiling. The solution were then cooled down and sodium nitrite (0.01 mol) was added, with stirring, until it was dissolved. The solution was cooled down by placing in an ice bath, and then it was acidified by hydrochloric acid (2 mL), and then water (3 mL) was added. By acidifying the solution, a powdery yellow precipitate of the diazonium salt was separated.

### 3.3. Sodium 2-[4-{2-[4-(dimethylamino)phenyl]-1-diazenyl} benzoylamino] Acetate **(3a)**

*N,N*-Dimethylaniline (0.01 mol) and glacial acetic acid (0.01 mol) were mixed. The solution of *N,N*-dimethylaniline acetate was added to suspension of hippuric acid, diazonium salt with stirring and acid-stable form of the dye was separated. A stiff paste was formed in 5–10 min and then sodium hydroxide (5 g) was added. The product was collected using saturated sodium chloride solution. The crude product was crystallized from water. Orange powder, decomposed >270°C yield is 81%. IR (KBr): *υ* = 3354, 1716 cm^−1^. ^1^H NMR (400 MHZ, DMSO-D_6_): 3.07 (s, 6H, 2CH_3_), 3.61 (d, 2H, *J* = 4.4 Hz, CH2), 6.84 (d, 2H, *J* = 8.9 Hz, ArH), 7.80–7.82 (m, 4H, ArH), 7.92 (broad, 1H, NH), 7.98 (d, 2H, *J* = 8.5, ArH) ppm. C_17_H_17_O_3_N_4_Na (348) calcd. C 58.65, H 4.88, N 16.08; found. C 58.54, H 4.92, N 16.18.

### 3.4. Sodium 2-({4-[2-(1-hydroxy-2-naphthyl)-1-diazenyl]benzoyl}amino) Acetate **(**3b**)**

2-Naphthol (0.01 mol) was dissolved in 5% sodium hydroxide solution (30 mL). The solution of 2-naphthol was added to suspension of hippuric acid diazonium salt with stirring, and base-stable form of the dye was separated. A stiff paste was formed in 5–10 min and then 10 mL of 10% acetic acid was added. The product was collected using saturated sodium chloride solution. The crude product was crystallized from water. The crude product was crystallized from water. Red powder, decomposed >236°C yield is 81%. IR (KBr): *υ* = 3469, 3364, 1714 cm^−1^. ^1^H NMR (400 MHz, DMSO-D_6_): 3.61 (d, 2H, *J* = 4.4 Hz, CH2), 6.88–8.63 (m, 12H, ArH, NH, OH) ppm. C_19_H_14_N_3_O_4_Na (371) calcd. C 61.46, H 3.77, N 11.32; found. C 61.73, H 3.66, N 11.09.

### 3.5. Sodium 2-({4-[2-(2-hydroxy-1-naphthyl)-1-diazenyl]benzoyl}amino) Acetate **(**3c**)**

1-Naphthol (0.01 mol) was dissolved in 5% sodium hydroxide solution (30 mL). The solution of 2-naphthol was added to suspension of hippuric acid diazonium salt with stirring and base-stable form of the dye was separated. A stiff paste was formed in 5–10 min, and then 10 mL of 10% acetic was added. The product was collected using saturated sodium chloride solution. The crude product was crystallized from water. Red powder, decomposed >259°C yield is 81%. IR (KBr): *υ* = 3477, 3355, 1710 cm^−1^. ^1^H NMR (400 MHz, DMSO-D_6_): 3.64 (d, 2H, *J* = 4.4 Hz, CH2), 6.90–8.89 (m, 12H, ArH, NH, OH) ppm,. C_19_H_14_N_3_O_4_Na (371) calcd. C 61.46, H 3.77, N 11.32; found. C 61.25, H 4.02, N 11.18.

### 3.6. General Procedure for Synthesis of Compounds **4a–4f**

A mixture of anhydrous sodium acetate (0.01 mol), 4-fluoro benzaldehyde or 4-trifluoromethoxy benzaldehyde (0.01 mol), sodium salt of azo dye **3a–3c** (0.01 mol), and acetic anhydride (40 mL) was heated with stirring until the mixture was transformed from an orange semisolid mass to a deep red liquid for 2–4 h. After cooling, the precipitated product was filtered and recrystallized in toluene [18].

### 3.7. General Procedure for Synthesis of Compounds **5a–5f**

To a solution of compounds **4a–4f** (2 mmol) in 50 mL of dry benzene was added 0.312 g (2 mmol) of 3,4-dithio-toluene and 0.2 mL of triethylamine. The mixture was stirred for 3 h at room temperature, then filtered, and washed with dry benzene. The residue was recrystallized from ethanol 96%.

### 3.8. Spectroscopic Data

#### 3.8.1. 4-Methyl-2-sulfanylphenyl(E)-2-[(4-{(E)-2-[4-(dimethylamino)phenyl]-1-diazenyl}benzoyl)amino]-3-(4-fluorophenyl)-2-propenethioate **(5a)**

Red powder; m.p. 293°C (decomposed); IR (KBr) *υ*: 3279 (NH), 1722 (C=O), 1662 (C=O) cm^−1^. ^1^H NMR (DMSO-D_6_, 400 MHz) *δ*: 2.23 (s, 3H, CH_3_), 3.14 (s, 6H, 2CH_3_), 4.35 (broad, 2H, NH and SH), 6.76–8.29 (m, 16H, vinyl and aromatic); ^13^C NMR (ppm): 21.1, 41.3, 112.7, 114.4, 123,9, 125.6, 126.5, 127.3, 127.6, 127.9, 129.1, 129.5, 129.8, 132.0, 134.7, 136.8, 138.0, 148.6, 152.4, 155.7, 156.1, 156.4, 171.4, 181.9; Anal. Calcd for C_31_H_27_N_4_O_2_FS_2_: C, 65.26; H, 4.74; N, 9.82. Found: C, 65.01; H, 4.53; N, 9.63.

#### 3.8.2. 4-Methyl-2-sulfanylphenyl(E)-2-[(4-{(E)-2-[4-(dimethylamino)phenyl]-1-diazenyl}benzoyl)amino]-3-[4-(trifluoromethoxy)phenyl]-2-propenethioate **(5b)**

Brown powder; m.p. 286°C (decomposed); IR (KBr) *υ*: 3352 (NH), 1716 (C=O), 1641 (C=O) cm^−1^. ^1^H NMR (DMSO-D_6_, 400 MHz) *δ*: 2.38 (s, 3H, CH_3_), 3.16 (s, 6H, 2CH_3_), 4.24 (broad, 2H, NH and SH), 6.77–8.37 (m, 16H, vinyl and aromatic). ^13^C NMR (ppm): 21.1, 41.3, 113.9, 115.7, 118.3, 123.7, 125.9, 126.2, 126.5, 127.3, 129.1, 129.4, 130.1, 131.9, 134.2, 134.7, 135.2, 138.1, 148.4, 150.2, 152.4, 155.6, 156.3, 171.2, 181.5; Anal. Calcd for C_32_H_27_N_4_O_3_F_3_S_2_: C, 63.58; H, 4.47; N, 9.27. Found: C, 63.22; H, 4.09; N, 8.97.

#### 3.8.3. 2-[(E)-2-(4-{[((E)-2-(4-fluorophenyl)-1-{[(4-methyl-2-sulfanylphenyl) sulfanyl] carbonyl}-1-ethenyl) amino]carbonyl}phenyl)-1-diazenyl]-1-naphthyl Acetate **(5c)**

Brown powder; m.p. 308°C (decomposed); IR (KBr) *υ*: 3241 (NH), 1765 (C=O), 1666 (C=O) cm^−1^. ^1^H NMR (DMSO-D_6_, 400 MHz) *δ*: 2.24 (s, 3H, CH_3_), 2.53 (s, 3H, CH_3_), 3.15 (broad, 2H, NH and SH), 7.29–8.77 (m, 17H, vinyl and aromatic); ^13^C NMR (ppm): 22.5, 41.2, 115.4, 118.1, 118.9, 124.1, 125.0, 125.5, 125.7, 126.1, 127.1, 127.5, 128.3, 129.0, 129.9, 130.4, 130.6, 131.8, 132.1, 132.9, 133.9, 135.2, 137.5, 138.9, 145.1, 151.7, 154,4, 157.1, 165.1, 170.9, 180.3; Anal. Calcd for C_35_H_26_N_3_O_4_FS_2_: C, 66.14; H, 4.09; N, 6.61. Found: C, 65.86; H, 4.33; N, 6.38.

#### 3.8.4. 2-((E)-2-{4-[({(E)-1-{[(4-Methyl-2-sulfanylphenyl) sulfanyl] carbonyl}-2-[4-(trifluoromethoxy)phenyl]-1-ethenyl}amino)carbonyl]phenyl}-1-diazenyl)-1-naphthyl Acetate **(5d)**

Red powder; m.p. 318°C (decomposed); IR (KBr) *υ*: 3281 (NH), 1741 (C=O), 1666 (C=O) cm^−1^. ^1^H NMR (DMSO-D_6_, 400 MHz) *δ*: 2.37 (s, 3H, CH_3_), 2.53 (s, 3H, CH_3_), 3.36 (s, 1H, SH), 7.27–9.02 (m, 19H, vinyl and aromatic, NH); ^13^C NMR (ppm): 22.5, 41.2, 114.9, 117.1, 118.3, 123.7, 125.1, 125.7, 126.2, 127.1, 127.4, 127.9, 128.5, 129.1, 129.8, 130.4, 130.9, 131.9, 132.2, 132.7, 134.1, 134.7, 136.1, 137.3, 138.4, 146.6, 148.1, 152.4, 155.9, 165.6, 170.8, 180.5; Anal. Calcd for C_36_H_26_N_3_O_5_F_3_S_2_: C, 61.63; H, 3.71; N, 5.99. Found: C, 61.44; H, 3.47; N, 6.07.

#### 3.8.5. 1-[(E)-2-(4-{[((E)-2-(4-fluorophenyl)-1-{[(4-methyl-2-sulfanylphenyl)sulfanyl] carbonyl}-1-ethenyl) amino]carbonyl}phenyl)-1-diazenyl]-2-naphthyl Acetate **(5e)**

Red powder; m.p. 325°C (decomposed); IR (KBr) *υ*: 3304 (NH), 1741 (C=O), 1666 (C=O) cm^−1^. ^1^H NMR (DMSO-D_6_, 400 MHz) *δ*: 2.20 (s, 3H, CH_3_), 2.65 (s, 3H, CH_3_), 3.34 (s, 1H, SH), 7.27–9.14 (m, 19H, vinyl and aromatic, NH); ^13^C NMR (ppm): 21.4, 41.3, 113.4, 117.1, 118.3, 123.4, 125.0, 125.5, 125.9, 126.1, 127.3, 127.5, 128.2, 129.0, 129.4, 130.4, 130.6, 131.9, 132.4, 132.8, 134.2, 135.2, 137.3, 138.1, 144.3, 152.3, 155.8, 156.3, 167.6, 171.5, 181.4; Anal. Calcd for C_35_H_26_N_3_O_4_FS_2_: C, 66.14; H, 4.09; N, 6.61. Found: C, 65.98; H, 4.45; N, 6.39.

#### 3.8.6. 1-((E)-2-{4-[({(E)-1-{[(4-Methyl-2-sulfanylphenyl) sulfanyl] carbonyl}-2-[4-(trifluoromethoxy)phenyl]-1-ethenyl}amino)carbonyl]phenyl}-1-diazenyl)-2-naphthyl Acetate **(5f)**

Red powder; m.p. 320°C (decomposed); IR (KBr) *υ*: 3308 (NH), 1721 (C=O), 1665 (C=O) cm^−1^. ^1^H NMR (DMSO-D_6_, 400 MHz) *δ*: 2.12 (s, 3H, CH_3_), 2.54 (s, 3H, CH_3_), 3.34 (s, 1H, SH), 7.24–9.01 (m, 19H, vinyl and aromatic, NH); ^13^C NMR (ppm): 21.4, 41.2, 115.7, 117.3, 118.3, 123.7, 125.4, 125.9, 126.2, 127.1, 127.3, 127.5, 128.0, 129.1, 129.4, 130.1, 130.3, 131.9, 132.2, 132.8, 134.1, 134.7, 135.2, 137.3, 138.1, 144.1, 148.4, 152.4, 156.3, 167.6, 171.2, 181.5; Anal. Calcd for C_36_H_26_N_3_O_5_F_3_S_2_: C, 61.63; H, 3.71; N, 5.99. Found: C, 61.92; H, 4.03; N, 6.11.

### 3.9. Tyrosinase Inhibition Assay

The spectrophotometric assay for tyrosinase was performed according to the method Ref 15. Briefly, all the synthesized compounds were screened for the diphenolase inhibitory activity of tyrosinase using L-DOPA as substrate. All the compounds were dissolved in DMSO. The final concentration of DMSO in the test solution was 2.0%. Phosphate buffer, pH=6.8, was used to dilute the DMSO stock solution of test compounds. Thirty units of mushroom tyrosinase (0.5 mg/mL) were first preincubated with the compounds, in 50 mM phosphate buffer (pH 6.8), for 10 min at 25°C. Then the L-DOPA (0.5 mM) was added to the reaction mixture, and the enzyme reaction was monitored by measuring the change in absorbance at 475 nm of formation of the L-DOPA chrome for 10 min. The measurement was performed in triplicate for each concentration and averaged before further calculation. IC_50_ value, a concentration giving 50% inhibition of tyrosinase activity, was determined by interpolation of the dose-response curves. The percent of inhibition of tyrosinase reaction was calculated as the following:

$$\begin{array}{cc} & \text{Inhibition}\;\left(\%\right)\;=\;\left[\text{B}-\frac{\text{S}}{\text{B}}\right]\;\times \;100. \end{array}$$

Here, the B and S are the absorbances for the blank and samples. All the experiments were carried out at least in triplicate, and the results represent means ± SEM (standard error of the mean). Kojic acid was used as reference standard inhibitors for comparison.

### 3.10. Inhibition of Melanin Production

Melanin production inhibition was ascertained by method of Wang et al. [19]. A total of 8 × 10^4^ cells were added to 60 mm plates and were incubated at 37°C in a CO_2_ incubator, then, 10 *μ*L test samples in DMSO were added to plates and were incubated for 72 hours at 37°C in a CO_2_ incubator. After washing with PBS, cells were destroyed with 1 mL of 1 N NaOH, and 200 *μ*L portions of raw cell extracts were moved to 96-well plates. Melanin production inhibition was determined by recording absorbance at 495 nm. The effects of test samples on melanin contents are stated as percent inhibitions of the value obtained in B16F10 mouse melanoma cells which were cultured with DMSO alone.

### 3.11. Cytotoxicity Assay

Cytotoxicity assays were performed using a microculture MTT method described by Han et al. [20]. A B16F10 mouse melanoma cell suspension was poured into a 96-well plate (10^3^ cells/well), and cells were allowed to completely stick to each other overnight. Test samples were then added to the plate and were incubated at 37°C for 72 h in a CO_2_ incubator. 20 *μ*L of MTT solution (2 mg/mL) was then added per well and incubated for 4 hours. Supernatant was then removed, and formazan was solubilized by adding 150 *μ*L DMSO to each well with mild shaking. Absorbance at 490 nm was recorded using an ELISA plate reader.

## 4. Conclusion

All synthesized azo dyes exhibited high tyrosinase inhibitory behavior. The results of mushroom tyrosinase inhibition assays indicate that the 4-trifluoromethoxy derivatives have high degrees of inhibition and 1-naphthol and 2-naphthol derivatives are better for tyrosinase inhibition than *N,N*-dimethylaniline derivatives. All synthesized azo compounds **5a–5f** showed the most potent mushroom tyrosinase inhibition, comparable to that of Kojic acid as reference standard inhibitors.

## Figures and Tables

**Table 1:** Structures, UV-Vis absorption, yields, and melting points of new sulfanyl azo compounds **5a–5f**.

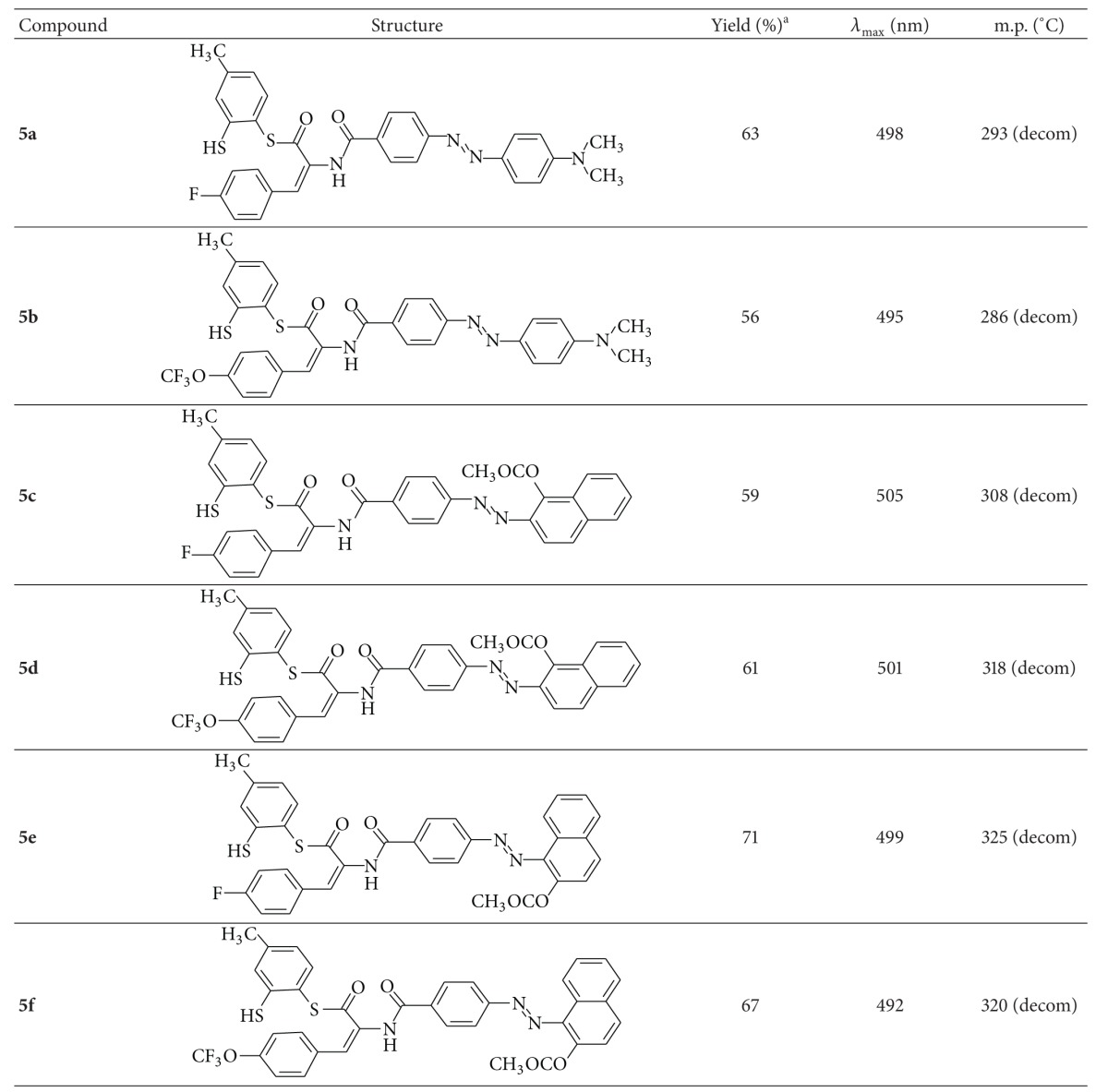

^a^Isolated yield.

**Table 2:** Tyrosinase inhibitory activities of the compounds **5a–5f**, as compared to the standard inhibitors.

Entry	Compound	IC_50_ ± SEM^a^ (*µ*M)
1	**5a**	4.39 ± 0.76
2	**5b**	3.86 ± 0.66
3	**5c**	2.68 ± 0.55
4	**5d**	2.48 ± 0.88
5	**5e**	1.98 ± 0.39
6	**5f**	1.71 ± 0.49
7	Kojic acid^b^	16.67 ± 0.52

^a^SEM is the standard error of the mean.

^b^Standard inhibitors of the enzyme tyrosinase.

**Table 3:** Melanin production and cytotoxicity.

Entry	Compound	Melanin production Inhibition (%)	Cytotoxicity cell viability (%)
1	**5a**	42.14 ± 0.75	82.15 ± 3.55
2	**5b**	40.98 ± 4.33	80.06 ± 1.89
3	**5c**	35.93 ± 3.11	83.15 ± 2.22
4	**5d**	37.50 ± 4.09	84.67 ± 5.38
5	**5e**	36.84 ± 1.44	81.91 ± 1.08
6	**5f**	34.74 ± 2.22	80.59 ± 2.65
7	Kojic acid^a^	17.28 ± 1.29^b^	81.68 ± 1.23^b^

^a^Standard inhibitors of the enzyme tyrosinase.

^b^Tested at 200 *µ*g/mL.

## References

[1] Lee H-S (2002). Tyrosinase inhibitors of *Pulsatilla cernua* root-derived materials. *Journal of Agricultural and Food Chemistry*.

[2] Masamoto Y, Ando H, Murata Y, Shimoishi Y, Tada M, Takahata K (2003). Mushroom tyrosinase inhibitory activity of esculetin isolated from seeds of *Euphorbia lathyris* L. *Bioscience, Biotechnology and Biochemistry*.

[3] Bandgar PB, Adsul LK, Chavan HV (2012). Synthesis, biological evaluation, and molecular docking of N-{3-[3-(9-methyl-9H-carbazol-3-yl)-acryloyl]-phenyl}-benzamide/amide derivatives as xanthine oxidase and tyrosinase inhibitors. *Bioorganic and Medicinal Chemistry*.

[4] Cho JC, Rho HS, Joo YH (2012). Depigmenting activities of kojic acid derivatives without tyrosinase inhibitory activities. *Bioorganic and Medicinal Chemistry Letters*.

[5] Ghani U, Ullah N (2010). New potent inhibitors of tyrosinase: novel clues to binding of 1,3,4-thiadiazole-2(3*H*)-thiones, 1,3,4-oxadiazole-2(3*H*)-thiones, 4-amino-1,2,4-triazole-5(4*H*)-thiones, and substituted hydrazides to the dicopper active site. *Bioorganic and Medicinal Chemistry*.

[6] Awad IMA, Osman AH, Aly AAM (2003). Heterocyclo-substituted sulfa drugs: part XII. Mercapto-S-azo-benzothiazol dyes and their metal complexes. *Phosphorus, Sulfur and Silicon and the Related Elements*.

[7] Akkurt B, Hamuryudan E (2008). Enhancement of solubility via esterification: synthesis and characterization of octakis (ester)-substituted phthalocyanines. *Dyes and Pigments*.

[8] Sevim AM, Arkan S, Koca A, Gül A (2012). Synthesis and spectroelectrochemistry of new phthalocyanines with ester functionalities. *Dyes and Pigments*.

[9] Khanmohammadi H, Darvishpour M (2009). New azo ligands containing azomethine groups in the pyridazine-based chain: aynthesis and characterization. *Dyes and Pigments*.

[10] Al-Rubaie AZ, Al-Fregi AA, Al-Jadaan SAS (2011). Synthesis of a new series of 2-(2-hydroxynaphthylazo)aryltellurium compounds. *Phosphorus, Sulfur and Silicon and the Related Elements*.

[11] Raposo MMM, Sousa AMRC, Fonseca AMC, Kirsch G (2005). Thienylpyrrole azo dyes: synthesis, solvatochromic and electrochemical properties. *Tetrahedron*.

[12] Karci F, Demirçali A, Şener I, Tilki T (2006). Synthesis of 4-amino-1*H*-benzo[4,5]imidazo[1,2-*H*]pyrimidin-2-one and its disperse azo dyes. Part 1: phenylazo derivatives. *Dyes and Pigments*.

[13] Yazdanbakhsh MR, Ghanadzadeh A, Moradi E (2007). Synthesis of some new azo dyes derived from 4-hydroxy coumarin and spectrometric determination of their acidic dissociation constants. *Journal of Molecular Liquids*.

[14] Zeng H, Lin ZP, Sartorelli AC (2004). Resistance to purine and pyrimidine nucleoside and nucleobase analogs by the human MDR1 transfected murine leukemia cell line L1210/VMDRC.06. *Biochemical Pharmacology*.

[15] Sharma P, Rane N, Gurram VK (2004). Synthesis and QSAR studies of pyrimido[4,5-*d*]pyrimidine-2,5-dione derivatives as potential antimicrobial agents. *Bioorganic and Medicinal Chemistry Letters*.

[16] Huang CQ, Wilcoxen KM, Grigoriadis DE, McCarthy JR, Chen C (2004). Design and synthesis of 3-(2-pyridyl)pyrazolo[1,5-*a*]pyrimidines as potent CRF1 receptor antagonists. *Bioorganic and Medicinal Chemistry Letters*.

[17] Abdel-Megeed MF, Badr BE, Azaam MM, El-Hiti GA (2012). Antimicrobial activities of a series of diphenyl(arylamino)(1-phenyl-3-(pyridin-2-Yl)-1H-pyrazol-4-Yl)methylphosphonates phosphorus. *Phosphorus, Sulfur, and Silicon and the Related Elements*.

[18] Hamidian H, Tagizadeh R, Fozooni S, Abbasalipour V, Taheri A, Namjou M (2013). Synthesis of novel azo compounds containing 5(4H)-oxazolone ring as potent tyrosinase inhibitors. *Bioorganic and Medicinal Chemistry*.

[19] Wang H-M, Chen C-Y, Chen C-Y (2010). (-)-*N*-Formylanonaine from *Michelia alba* as a human tyrosinase inhibitor and antioxidant. *Bioorganic and Medicinal Chemistry*.

[20] Han J, Ma I, Hendzel MJ, Allalunis-Turner J (2009). The cytotoxicity of *γ*-secretase inhibitor I to breast cancer cells is mediated by proteasome inhibition, not by *γ*-secretase inhibition. *Breast Cancer Research*.

